# Drug Repositioning in the Mirror of Patenting: Surveying and Mining Uncharted Territory

**DOI:** 10.3389/fphar.2017.00927

**Published:** 2017-12-15

**Authors:** Hermann A. M. Mucke

**Affiliations:** H. M. Pharma Consultancy, Wien, Austria

**Keywords:** chemistry, data mining, drug repositioning, expert systems, patents as topic

## A multimodal approach to the re-development of drugs

Drug repositioning—the investigation, development and use of active pharmaceutical ingredients for a therapeutic class that is different from the original one—is much more than a “recycling” of known drugs or drug candidates (Oprea and Mestres, 2012). Quite the opposite, it creates novel insights that are of additional scientific and public health interest. Exploiting the fact that very few compounds act on only one molecular target is just one aspect; drug repositioning can also use drug targets as a starting point to screen compound libraries. Nowadays it goes far beyond serendipity, making use of the newest insights in chemical genomics (Bisson, 2012), computational biology (Hodos et al., 2016; Li et al., 2016), systems medicine (Mei et al., 2016), and text mining (Tari and Patel, 2014) to utilize pharmacological activities (known or newly identified) in more ways than had been originally envisaged. This approach extends to biotechnology products, and even vaccines (Veljkovic and Paessler, 2016).

The constantly growing number of repositioning-related peer review papers in PubMed reflects the growing scientific interest in the subject. For 2016, a search for papers indexed under the MeSH term “drug repositioning” or the keyword “drug repurposing” returned 306 hits. Considering that many relevant contributions are not being indexed in this way, and do not contain “drug repurposing” in their titles or abstracts, this captures only part of the actual publishing activity even as reflected in PubMed-listed journals.

## Patenting in drug repositioning projects

Drug repositioning offers more immediate value than developing a new chemical entity. Re-developing an active compound that is already marketed, or at least has some accessible preclinical or clinical data, is associated with a substantially reduced risk of failure that may be due to safety, resulting in improved success rates (Caban et al., 2017). Depending on circumstances, the path toward regulatory approval can be shortened, and savings in terms of time to market and cost can be realized. Although there are many issues to consider in the commercial valuation of a drug re-development project, obtaining intellectual property for the new use (frequently combined with a new formulation, which may involve a different route of administration) will always be a paramount factor (Sternitzke, 2014).

## Patents as an information source for drug repositioning R&D

There is more to patenting than just establishing intellectual property. Drug-related patents are a source of pharmacological and developmental information that offers an orthogonal perspective on research and development activity in the field: while peer reviewed journals aim at publishing what has scientific novelty, patenting primarily reflects what inventors and patent assignees deem to be of relatively immediate applicability. Its requirements for novelty and non-obviousness are not academic but practical ones. To obtain a complete perspective of drug repositioning activity, it would be extremely worthwhile to investigate patent documents as a complement to peer reviewed publishing.

This is not a trivial task because no ontology or keyword pattern exists that would allow a targeted search for “novel medical use” patents. In effect, the abstracts of all patents in the pharmaceutical category (and in many cases, the full texts) have to be examined for such content. For international disclosures published under the Patent Convention Treaty, this amounts to 250–300 documents per week that need to be screened.

## What studying drug repositioning patents can offer

We have shown earlier that source and target indications of drug repurposing, as reflected in international patent applications published during the years 2011–2014, are not randomly distributed in therapeutic space but follow preferred vector patterns (Mucke and Mucke, 2015). Unexpected findings included frequent secondary use claims for oncology agents to treat noninfectious respiratory diseases, and for cardiovascular agents to treat neurological conditions.

A superficial look at the most recent 3-year period between October 2014 and September 2017 confirms that patenting activity in this field continues to be substantial, and is growing. In each quarter during this interval, between 17 and 39 international patent applications claiming drug repositioning matters have been published, to a three-year total of 329 documents—on average, about two per week. Just as with the respective counts in PubMed, these are minimum figures because the repositioning-related content of a patent is sometimes not apparent from its title or abstract.

Some of these patents provide extremely interesting insights into drug re-development work in industry and academia that are not published elsewhere, or are published only later. Arbitrarily chosen examples include WO/2014/164667 reporting that entacapone, a COMT inhibitor used in Parkinson's disease, inhibits Dengue and West Nile virus proteases; WO/2015/189650 claiming the antithrombotic clopidogrel for benign prostate hyperplasia; claims for the X-ray contrast agents iopamidol and iohexol for treating influenza (WO/2015/116861) and Ebola virus (WO/2016/054658) infections; and the potential utility of gamma secretase inhibitors, notorious for their efficacy failures in Alzheimer's disease clinical trials, for multiple myeloma and leukemias (WO/2017/019496).

Many of these documents do not provide the reader with the type and extent of data support that would be expected from a peer-reviewed paper. But then full data sharing is not their primary purpose (“just enough” is the rule, so that later work is not preempted), and what they do disclose is sometimes tantalizing.

## Strategies for repositioning patent identification and analysis

There are few firm rules to define how the descriptive part of a patent document has to be written. Even just to find out if a given pharmaceutical patent has drug repositioning content can seem difficult to the novice if the document uses the legalistic and repetitive language that patent attorneys often impose on its actual content – for good reasons, discussion of which is beyond this article's scope. However, with practice the required information is relatively easily found, and the scientific information extracted, using manual searches.

Algorithmic text mining of patent documents for information relevant for drug repositioning remains an unmet challenge. In principle, most approaches that have been developed for mining the peer reviewed biomedical literature for this purpose should also be applicable to patent documents, with relatively few modifications. Integrated strategies that combine keyword searches with genetic regulation and disease phenotypes (Jang et al., 2017; Sun et al., 2017) seem most promising. However, efficacy and reliability of such approaches have never been systematically evaluated.

Extracting chemical information that is presented as Markush formulas is a much more complicated matter. Academic papers use this mode of summarizing many related molecules sparingly, by showing only their common core moiety and then inserting placeholders for substituents or substructures, whose meaning is verbally or graphically defined in the accompanying text. In contrast, pharmaceutical patenting makes extensive use of Markush structures, which at present cannot be algorithmically parsed for known compounds that might be included in the multitude of possibilities covered by the generic structural formula. Interesting approaches to this challenge exist (Deng et al., 2011) but are rarely found in the biomedical literature. They will need much more development before they can provide more than assistance to the pharmacologist or medicinal chemist who is interested in finding out if a particular new use for known compounds is already patented, or who is searching for new opportunities for a given molecule.

## Summary

Drug repositioning researchers are well advised not to ignore what patent documents have to offer, even if their format might be different, but doing so requires some experience. Developing improved expert systems that assist researchers in the identification and extraction of relevant information is an important task that could eventually result in algorithms that can run unattended. However, this needs not only programming but also calibration and validation, which does not seem to have been attempted yet. Systematic collections of drug repositioning patent documents could provide a most valuable reference standard in this context.

## Author contributions

The author confirms being the sole contributor of this work and approved it for publication.

### Conflict of interest statement

The author declares that the research was conducted in the absence of any commercial or financial relationships that could be construed as a potential conflict of interest.
